# Angiogenesis in Spinal Cord Injury: Progress and Treatment

**DOI:** 10.7759/cureus.25475

**Published:** 2022-05-30

**Authors:** Konstantinos Tsivelekas, Dimitrios Stergios Evangelopoulos, Dimitrios Pallis, Ioannis S Benetos, Stamatios A Papadakis, John Vlamis, Spyros G Pneumaticos

**Affiliations:** 1 Second Department of Orthopaedics, KAT General Hospital, Athens, GRC; 2 Third Department of Orthopaedics, National and Kapodistrian University of Athens School of Medicine, KAT General Hospital, Athens, GRC

**Keywords:** spinal cord regeneration, angiogenetic factor, angiogenesis, revascularization, spinal cord injury

## Abstract

Traumatic spinal cord injury (SCI) provokes the onset of an intricate pathological process. Initial primary injury ruptures local micro-neuro-vascularcomplex triggering the commencement of multi-factorial secondary sequences which exert significant influence on neurological deterioration progress. Stimulating by local ischemia, neovascularization pathways emerge to provide neuroprotection and improve functional recovery. Although angiogenetic processes are prompted, newly formed vascular system is frequently inadequate to distribute sufficient blood supply and improve axonal recovery. Several treatment interventions have been endeavored to achieve the optimal conditions in SCI microenvironment, enhancing angiogenesis and improve functional recovery. In this study we review the revascularization pathogenesis and importance within the secondary processes and condense the proangiogenic influence of several angiogenetic-targeted treatment interventions.

## Introduction and background

Spinal cord injuries (SCI) account for a worldwide incidence estimated from 250,000 to 500,000 per year, while they mostly occur during high energy injuries such as vehicle accidents, falls from height, gunshots, etc. [1]. Frequently followed by significant lesions, including compressions, ruptures, and fractures, spinal cord injuries result in a range of neurological symptoms depending on the level and severity of the injury. The neurological deficit is generally determined within 72 h after SCI, while post-traumatic processes can be divided into acute, subacute, intermediate, and chronic (Table 1) [2,3]. Almost half of the patients will not regain their everyday functionality, whereas the majority of them experience chronic types of pain [2,4].

**Table 1:** Post-traumatic progress time distribution

Stage of Spinal cord injury	Time elapsed since precipitation
Acute	< 48 hours
Sub-acute	2 days – 2 weeks
Intermediate	2 weeks – 3 months
Chronic	>3 months

Primary mechanical injury triggers the onset of a secondary multifactorial process [2]. Over the last decades, extensive research has been performed regarding the progressive damage on the lesion site, including vascular disorders, inflammatory process, demyelination, and cell apoptosis, resulting in glial scar and cavity formation and having a significant impact on axon regeneration and functional recovery [5]. Vascular integrity and adequacy are determining, providing a propitious regeneration microenvironment on the lesion site.

Although endogenous angiogenesis is triggered by SCI and the ensuing ischemia, the local vascular response is usually insufficient [6]. Hence, the meticulous comprehension of the SCI microenvironment is significant, in achieving the optimal conditions for axonal and functional recovery. Several pieces of research and various pre-clinical approaches have focused on regulating the vascular response following SCI, obstructing secondary processes, and contributing to the development of an organized and properly functional vascular system. Angiogenetic factors administration, gene modulation, and multiple treatment interventions stimulate revascularization on the injury site, providing promising results in SCI management [7].

In this study, a review of the literature determined the crucial role of the secondary vascular process within SCI providing an overview of vascular impairment within SCI, angiogenetic response on the lesion site, and conceivable proangiogenic interventions, promoting local angiogenesis and functional recovery.

## Review

Injury classification

Several classification algorithms have been proposed to ensure the most optimal conditions and the proper management of SCI, considering predominantly anatomical (skeletal level of injury) as well as functional-neurological points of criteria [8,9]. In order to determine the accurate level of spinal cord lesion, regarding the neurological level of SCI, the International Standards for Neurological Classification of Spinal Cord Injury (ISNCSCI) developed the American Spinal Injuries Association (ASIA) score. Since 1982, ASIA defined the “key muscles and dermatomes”, aiming to the particular assessment and documentation of SCI through the ASIA motor-sensory-impairment scale. ASIA or AIS scale is able to provide an integrated evaluation of the significance of the neurological distinction (complete to incomplete), providing essential management documentation while often assembling crucial prognostic details [10].

Spinal cord injury pathophysiology

Primary Injury

**Figure 1:**
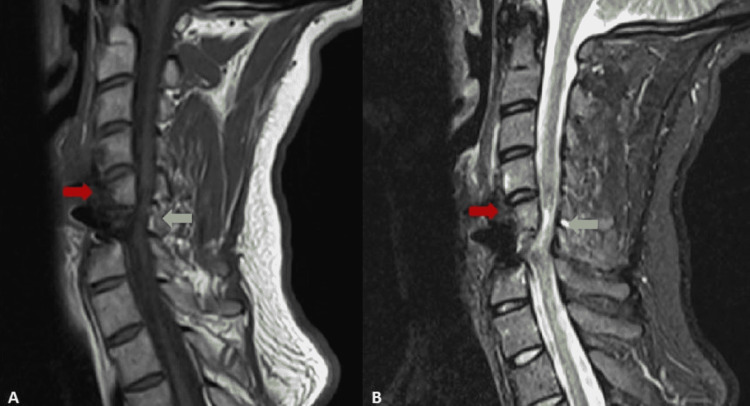
Sagittal views of the cervical spine fracture A) Sagittal T1 view of cervical spine fracture B) Sagittal T2 view of the cervical spine fracture. The red arrow is showing the C6 vertebrae fracture. The green arrow reveals the concomitant compression of the spinal cord.

Direct or persistent compression forces exerted among the canal seem to mostly insult spinal cord integrity. The primary stage of SCI consists of mechanical forces, shrill damage, and strain to the spinal cord and the surrounded neurovascular complex through fracture fragments, dislocations, strain, compression, and/or torsion forces (Figure 1) [11]. Accompanied hematomas constitute a presumable compression threat of the spinal cord, as bleeding erupts shortly after SCI, followed by local ischemic infraction and hypoxia [12]. Primary lesion harmfully affects gray matter, neurons, and oligodendrocytes, which essentially mediate neuronal transmission, whereas vascular damage, including blood spinal cord barrier (BSCB), augments inflammatory cell infiltration [13]. Furthermore, edema and macrophage accumulation on the lesion site aggravates distortion in neuronal transmission. Initial injury triggers a consequent secondary "cascade" contributing to further deterioration of the damaged spinal cord and consequent neurological impairment [14].

Secondary Injury

The secondary injury was firstly described by Allen in 1911 while studying SCI in dogs [15]. Secondary injury onsets shorty after SCI and is able to last for a week to months, concerning a multifaceted pathological self-destruction process of the spinal cord at cellular, biochemical and molecular levels [5]. Divided into acute, sub-acute, and chronic secondary mechanisms of injury, encompass a handful of perturbations containing vascular damage, ionic imbalance, excitotoxicity (neurotransmitter accumulation), free radical development, calcium inundation, inflammatory processes, edema, and necrotic cell death [16]. Progressively, apoptosis, axial demyelination, Wallerian deterioration/degeneration, matrix remodeling, and glial scar formation on the injury site consist of the sub-acute stage, until the lastly developed axonal dieback, cystic cavity accretion, and glial scar maturation on the chronic phase [17,18].

Vascular Response After SCI

The vascular system consists of a well-architectured and organized structure providing a supportive environment to the nervous system [19]. Initial spinal cord injury results in local vascular impairment and BSCB deterioration, intensifying vascular permeability, while the secondary injury progress demonstrates deterrent conditions for regeneration and functional recovery [20]. Mechanical trauma consequences on disruption of perivascular basement membrane (BM) and detachment of extracellular matrix (ECM), leading to a huge loss of endothelial cells (EC) and EC apoptosis, instigated by the ensuing ischemia [21,22]. Disorders in BM structure intensify the inflammatory response outspread, impairing cell death in the subacute stage, while the accompanied hemorrhage aggravates axonal breakdown [23]. In addition, mechanical shear induces disruption of BSCB, occurring in the very first hours of the injury. Pro-inflammatory cytokines (interleukin{IL}-1β, tumor necrosis factor {TNF}a), metalloproteinase (MMPS), and vasoactive agents exacerbate vascular permeability, contributing to inflammatory infiltrate and secondary injury process on the injury site [6,24,25]. 

Endogenous Angiogenesis

Angiogenesis consists of the fundamental form of vessel evolvement in the injury site after SCI, prompted by local hypoxia and proangiogenic substances, and includes chiefly three formation mechanisms: vasculogenesis, splitting angiogenesis, and sprouting angiogenesis [26,27]. Vasculogenesis concerns the “de novo” vessel formation from precursor endothelial cells or angioblasts, generating predominantly among embryogenesis [28]. Regarding SCI, new vessels proceed through the pre-existing vascular system (angiogenesis), separated into two main processes: firstly, sprouting angiogenesis where ECM restructures and reorganizes providing a propitious microenvironment for EC migration, escalation, tube formation, and lastly configuration of new sprouts and intussusception (or splitting angiogenesis), accomplished by old-vessel splitting [27,29].

Angiogenesis and EC remodeling and regeneration are stimulated by local ischemia and augmented by miscellaneous molecules through various signaling pathways [30,31]. Endothelial cell augmentation is provided by several transcription agents including Sox17, Foxo1, FoxM1, Atf3, and HIF-1a, exerting influence on the sprouting angiogenesis process, which is additionally affected by Mef2 factors/agents [32,33]. Vascular endothelial growth factor (VEGF), one of the most identified angiogenic components in blood vessel formation and regeneration, engages angiogenic pathways in both regular and pathological circumstances [34]. Expression of HIF-1a is increased through PI3K-Akt signaling set-off and induces VEFG production, contributing to the angiogenetic process, whereas prevention of the PI3K-Akt and mTOR signaling pathways prompt Foxo1 expression and instigate EC curvature [35,36]. Notch signaling conduces to sprouting and splitting procedure management through Dll4 expression in tip cells [37].

The secondary injury process accelerates rapidly following SCI, hence endogenous angiogenesis is frequently inadequate to confront the progressive local ischemia and cell death. New vessels ephemeral enhance within two weeks after SCI providing an early-novel scaffold for axonal renascence and remodeling, however, accompanied BSCB disruption often lays in peril vessel vulnerability [37]. Additionally, the development of the recently formed vessels into a well-structured and functional vascular system usually fails due to their anatomic features and lack of connections with local neurons, vascular mural, and glial cells, resulting in poor branching germination [6,23].

Several studies have proved the beneficial role of sufficient capillary blood flow, angiogenesis, and BSCB probity to ensure the distinguished conditions for tissue survival and functional regeneration [38,39]. Significant interaction among vessel regrowth and nerve recondition has been demonstrated concerning both salutary and repellent evidence, including Slits, Nogo, Semaphorins, Ephrins, VEGF, neurotrophins (NGF, NT-3), vascular cell types (vSMCs), astrocytes, microglia, and oligodendrocytes [19,40-42]. By and large, revascularization is crucial in SCI rehabilitation. Interventions targeting the harmed vascular system by providing blood supply adequacy, triggering angiogenesis, and ensuring BSCB integrity and vascular decency, are capable of potentially diminishing secondary progression and promoting axonal guidance and functional recovery following SCI.

Revascularization treatment interventions following SCI

Development of vascular response and augmentation of the angiogenetic process within SCI conducts the delivery of proangiogenic factors, gene regulation, and several vascular interventions. However, modulation of adjustable and controlled angiogenesis prevails the vast research confrontation. Increased microvascular permeability carries the risk of spreading the lesion through leukocyte infiltration, while immoderate VEGF expression has been implicated in tumor formation [43].

(i) Proangiogenic factor administration: Blood vessel formation, as well as, ECs migration and proliferation is significantly affected by vascular endothelial growth factor (VEGF) [34]. Isolated or combined VEGF administration and its isoform (VEGF-A165, 121, 189) provided significant post-traumatic enhancing recovery and neuroprotection in numerous studies [44,45]. Delivery of modified zinc protein transcription factor (ZFP) activates all isoforms of VEGF-A, whereas a combination of VEGF with platelet-derived growth factor (PDGF)/fibroblast growth factor-2 (FGF2)/Angiopoietin (ANG1) improved blood vessel density, abated BSCB permeability and enhance blood supply [46-48].

Several hormones, enzymes, or substances such as melatonin and estrogen, have been shown to detect an angiogenetic influence in SCI management [49]. Chondroitinase ABC (ChABC) provides axonal remodeling and regeneration by triggering revascularization. Studies have presented the shrinking of extracellular chondroitin sulfate proteoglycans (CSPG) by ChABC, stimulating neoangiogenesis and protecting vessel BM [50,51]. Additionally, MMPs, flufenamic acid (FFA) or MMP-8 inhibitor (MMP-81), and granulocyte colony-stimulating factor (G-CSF), provide a permissive environment for local revascularization and BSCB disruption deterrence [52,53].

(ii) Gene modulation: Neuroprotection and functional recovery through several genetic pathways has been observed in assorted experimental studies, concerning the reciprocation of genetic perturbation in proangiogenic factors expression [54]. Kumar et al. assessed the transient potential channel protein (TRPV4) fluctuation and increase following SCI, providing the statement of the detrimental repercussion of TRPV4 activation in endothelial cell damage, inflammation progress, and rehabilitation/functional recovery [55]. Diminution of UTX (Ubiquitously Transcribed tetratricopeptide repeat on chromosome X), a histone H3K27 demethylase, which is significantly increased after SCI, augments EC migration and tubule/tube formation/genesis and enhances epigenetically the vascular remodeling and functional retrieval through miR-24pathway [56]. Blocking in proangiogenic microRNAs expression outcomes in inflammatory impediment and proceeds vascular growth. Knockdown of PTP1B and EFNA3 through miR-210 delivery, as well as SPRED1 AND PIK3R2 crush prompted by Agomir-126/miR-126 administration, operated in revascularization and functional recovery [57,58].

(iii) Cell-based therapeutic strategies: Stem cell transplantation erupted as a persuasive path in both degenerative and traumatic disorders owing to their immanent differentiation diversity and providing auspicious treatment options [59]. Mesenchymal stem cells (MSCs) originating from the umbilical cord, adipose tissue, amnion, and bone marrow have been observed to promote BSCB restoration and enhance revascularization on the lesion site [60,61]. Exosomes or extracellular vesicles originating from MSC include secreted or paracrine-secreted proangiogenic factors, emulating MSCs' endeavor in revascularization [40].

Neural stem/progenitor cells (NS/PCs) or neural stem cells' potential differentiation into neurons, astrocytes, and oligodendrocytes provided an augmented angiogenetic impact on the damaged spinal cord [62,63]. Stimulated by VEGF secretion, NSCs transplantation or co-implantation with ECs offers a prosperous microenvironment for ministrant vascular regrowth and BSCB maintenance/conservation, whereas the astrocytic module of NCS can also merge with endogenous astrocytes through migration providing further BSCB integrity [64-66]. Additionally, numerous cells including HUVECs, pericytes, and CD133+ blood cells have been assessed due to their proangiogenic effect on SCI [67,68].

(iv) Other angiogenetic administrators: Natural or synthetic biomaterials, including hyaluronic acid (HA), collagen, fibrin, Poly-L-lactic acid (PLL), poly-lactic-co-glycolic (PLGA) have been observed to enhance revascularization following SCI [7]. Furthermore, physically stimulated treatments in mice models provided an intrinsic angiogenic effect and promoted EC proliferation and BSCB maintenance on the lesion site, comprising hypoxia-induced angiogenesis (chronic mild hypoxia) and water treadmill training [69,70].

An abundance of treatment interventions has been endeavored, utilizing their angiogenetic influence on lesion sites as illustrated in Table 2.

**Table 2:** Angiogenesis enhance administrators. bFGF = basic fibroblast growth factor, Ang-1 = angiopoietin, FGF2 = fibroblast growth factor 2, ChABC = chondroitinase ABC, FFA = flufenamic acid, MMP-8 = Matrix, Metalloproteinase-8, G-CSF = Granulocyte colony-stimulating factor, HGF = hepatocyte growth factor, TRPV4 = transient receptor potential vanilloid type 4, UTX = Ubiquitously Transcribed tetratricopeptide repeat on chromosome X, miR-210 = micro-RNA 210, miR-126 = MicroRNA-126, hAMSCs = Human Amniotic Mesenchymal Stem Cells, hADSCs = human adipose tissue-derived mesenchymal stromal cells, NS/PCs = neural stem/progenitor cells, hiPSCs = human-induced pluripotent stem cells, ECs = endothelial cells, NPCs = neural progenitor cells, FPSS = fibrous porous silk scaffold, HUVECs = human umbilical vein endothelial cells, ADAMTS13 = ADAM metallopeptidase with thrombospondin type 1 motif 13, AFG = aligned fibrin hydrogel, HA = hyaluronic acid, PLL= poly-L-lysine, antiNgR = nogo-66 receptor antibody, CS = collagen scaffold, CBD-VEGF = constructed protein, collagen-binding VEGF Kitamura et al. [54]; Kumagai et al. [64]; Rauch et el. [65]; Sasaki et al. [71]; Kang et al. [72]; Herrera et al. [46]; Wei et al. [73]; Kawabe et al. [52]; Nori et al. [63]; Fujioka et al. [68]; Zhou et al. [62]; Milbreta el al. [51]; Ujigo et al. [58]; Wu et al. [49]; Hu at el. [57]; Badner et al. [67]; Samantaray et al. [74]; Yu et al. [75]; Zhou et al. [61]; Jing et al. [76]; Halder et al. [70]; Kumar et al. [53]; Yao et al. [77]; Wang et al. [78]; Yao et al. [79]; Ni et al. [56]; Kumar et al. [55]; Zhong et al. [80]

Author	Method	Results
Kitamura et al. 2007	Delivery of HGF gene	Increased neuron survival, promoted angiogenesis and functional recovery
Kumagai et al. 2009	Transplantation of NS/PCs	Stimulated angiogenesis, axonal volume and remyelination, promoted locomotor recovery
Rauch et el. 2009	Administration of a co culture of ECs and NPCs	Induced angiogenesis
Sasaki et al. 2009	Administration of CD133^+^ cells	Increased VEGF expression, promoted angiogenesis, axonal regeneration, functional recovery
Kang et al. 2010	Delivery of FGF2	Increased spinal cord blood flow, improved vessel density
Herrera et al. 2010	Co-delivery of VEGF and Ang-1	Vascular stabilization, improved locomotor function
Wei et al. 2010	Administration of HA-based hydrogels modified with PLL and antiNgR	Increased angiogenesis and prevented glial scar formation
Kawabe et al. 2011	Delivery of G-CSF	Increased angiogenic cytokines, improved locomotor function
Nori et al. 2011	Transplantation of hiPSCs	Stimulated angiogenesis, axonal volume and remyelination, promoted locomotor recovery
Fujioka et al. 2012	Transplantation of CD133^+^ cells+ application of a magnetic field	Improved neurological recovery
Zhou et al. 2013	Transplantation of hADSCs[comparison with hAMSCs]	hADSCs led to higher levels of VEGF and HGF and improved functional recovery
Milbreta et al. 2014	Delivery of ChABC	Increased revascularization
Ujigo et al. 2014	Delivery of miR-210	Increased angiogenesis, and improved locomotor function, decreased cell apoptosis
Wu et al. 2014	Delivery of melatonin	Decreased BSCB permeability,cell apoptosis, consolidation of the microcirculation
Hu at el. 2015	Delivery of miR-126	Promoted angiogenesis, reduced vascular inflammation, improved vascularity
Badner et al. 2016	Delivery of Human Brain Stromal Cells	Diminished BSCB permeability, and hemorrhage, improved neurological outcome
Samantaray et al. 2016 , Ni et al. 2018	Delivery of estrogens	Increased angiogenic factors, improved microvessel growth and locomotor function
Yu et al. 2016b	Co-delivery of VEGF, ang-1 and bFGF	Increased expression of angiogenic factors, promoted angiogenesis and neurogenesis, improved neurological function.
Zhou et al. 2016	Transplantation of hAMSCs	Increased levels of VEGF, promoted axonal regeneration, improved functional recovery
Jing et al. 2017	Delivery of melatonin	Increased circulation in the spinal cord, improved neurological outcome
Halder et al. 2018	Chronic mild hypoxia	Promoted angiogenesis and endothelial multiplication
Kumar et al. 2018	Delivery of MMP-8 inhibitor	Decreased inflammation, BSCB damage and cell impairment
Yao et al. 2018a	Administration of AFG	Supported angiogenesis and axonal regeneration
Wang et al. 2018	Implantation of CS targeted with CBD-VEGF	Promoted angiogenesis, axonal regeneration, enhanced the microenvironment
Yao et al. 2018b	Delivery of FFA	Prevented capillary fragmentation, induced angiogenesis, reduced hemorrhage and BSCB disruption
Ni et al. 2019	Knockdown of UTX	Increased vascular regeneration and promoted neurological recovery
Kumar et al. 2020	Delivery of TRPV4 antagonist	Decreased inflammation, preserved BSCB, reduced scarring, improved functional outcome
Zhong et al. 2020	Administration of cocultured FPSS with ADAMTS13-overexpressing HUVECs	Promoted neovascularization, microvascular formation and functional recovery

## Conclusions

Spinal cord injuries signify the onset of inherent pathological complex process. There is peremptory necessity for an extensive knowledge of all the intrinsic procedure occurring within the initial injury, so that the optimal conditions for the proper treatment and recovery will be ensured. Vascular disruption, following SCI, comes up with a dominant consistency, regarding the progression of the injury. Improved technological methods and multiple studies have been developed to ensure an adequate blood supply at the site of injury and develop well-functioned vascular system providing promising outcomes, however many aspects of both pathophysiological and angiogenetic processes remain unspecified. Presumably, integration of proangiogenic strategies could provide braced outcomes, although further research is essential.
